# Case-only analysis in small studies of predictive biomarkers

**DOI:** 10.1038/s41598-025-96904-9

**Published:** 2025-04-16

**Authors:** M. Hauptmann, V. H. Nguyen, L. Sollfrank, S. C. Linn, K. Jóźwiak

**Affiliations:** 1 Brandenburg Medical School Theodor Fontane, Institute of Biostatistics and Registry Research, Fehrbelliner Straße 39, 16816 Neuruppin, Germany; 2 Division of Molecular Pathology, The Netherlands Cancer Institute, Amsterdam, The Netherlands; 3 Department of Medical Oncology, The Netherlands Cancer Institute, Amsterdam, The Netherlands; 4 Department of Pathology, University Medical Center, Utrecht, The Netherlands; 5 Present Address: Leibniz Centre for Agricultural Landscape Research (ZALF), Müncheberg, Germany

**Keywords:** Biomarker-treatment interaction, Case-only analysis, Firth’s penalized maximum likelihood, Treatment heterogeneity, Biomarkers, Clinical trials, Predictive markers

## Abstract

Characteristics of tumors and patients can be used as predictive biomarkers to guide treatment choice. Although many potential biomarkers are evaluated each year, only few will eventually be used since evidence is usually based on small studies leading to inconclusive results. Such data are often analyzed with Cox proportional hazards regression using a multiplicative interaction term between biomarker and treatment, with insufficient power and possibly biased results. Instead of analyzing patients who do (cases) and do not experience (non-cases) the survival event of interest, case-only analysis with logistic regression has been proposed, however with unknown small sample properties. We evaluated the performance of case-only analysis with bias-eliminating Firth correction and confidence intervals obtained with a profile likelihood method in a simulation study tailored to breast cancer. Our results show that this approach is generally inferior to the full cohort analysis but has acceptable properties when the marker is protective or null among patients treated with the standard treatment, the event rate is low (e.g., a rare event and a protective marker) and treatment assignment is independent of the marker level (e.g., in randomized studies). In such situations, the case-only design offers substantial cost savings. However, the model is sensitive to these assumptions.

## Introduction

Personalized medicine aims to find effective treatments for selected individuals. In oncology, for example, the selection can be based on the genetics of the tumor or healthy tissue, the tumor (immune) environment, lifestyle or comorbidities of patients^1^. These characteristics, called predictive biomarkers, may predict a patient’s response to a particular treatment, i.e., they indicate how one should be treated^2^. However, even though many candidate biomarkers are discovered in laboratories, for instance, by screens on cells, rodents or humans, only few end up being used in clinical practice. One reason for this may be the rigorous process biomarkers have to go through, culminating in a randomized clinical trial as the last step. Such a trial generally requires a large number of patients, access to patient specimen, and standardized assays for biomarker measurement. Unfortunately, these studies are often prohibitively expensive or suitable patients with appropriate tissue samples are scarce, so that these early clinical studies are often too small. This reduces power and causes small sample bias when suboptimal statistical methods are applied, which may lead to abandoning a promising biomarker.

A commonly used statistical method for evaluating a binary predictive biomarker is the Cox proportional hazards regression for failure time data^3^ with a multiplicative interaction term between biomarker and treatment. The interaction term indicates whether the relative effect of an experimental treatment in comparison to a control treatment differs by biomarker level^4–6^. In our earlier work^7^, we show that in particular settings specific to studies on predictive biomarkers, this method yields biased results and overestimates the standard error of the interaction term for cohort sizes under 600 patients. We also show that bias is reduced when the score function of the Cox model is modified with a Firth correction^8^ and confidence intervals (CIs) are obtained with a profile likelihood (PL) approach. However, results of studies with less than 400 patients rarely have sufficient power to detect interaction between biomarker and treatment. Thus, there is a need for the development of new statistical methods or the adaptation of standard methods for small studies of predictive biomarkers.

It has been shown that the interaction coefficient and treatment effects in biomarker subgroups can be estimated in the subset of patients who experience the event of interest, i.e., cases only^9–11^. The estimation is unbiased if the event rate is low, censoring is non-informative, and the biomarker level and treatment assignment are independent. With such a design, a simple logistic model can be used instead of a Cox model. The case-only design has been proposed more than a decade ago but has only rarely been applied in biomarker studies^12,13^. Epidemiologists, on the other hand, have used it for a long time to evaluate gene-environment or gene-gene interactions on binary outcomes^14–16^. In such studies, a case-only design is being used as an alternative to the case-control design since it obviates the need for genetic assays in non-case subjects and even provides a more efficient estimate of the interaction coefficient under the assumption of independence between the genetic and the environmental factors. Note that the assumption has to be made in observational studies but it is fulfilled by design when treatment is randomized.

Here, we performed a simulation study and designed it using results from real clinical studies on breast cancer (BC)^17–21^. Three of the studies were randomized controlled trials and two were observational series of patients. All studies had used archived specimens for biomarker measurements and evaluated interactions with either chemotherapy or endocrine therapy on risk of BC relapse or death due to any cause (recurrence-free survival, disease-free survival) or death due to BC (breast cancer-specific survival). The simulated data was analyzed using cases only with a logistic model corrected with the bias-eliminating approach developed by Firth^8^ and a CI calculated using a PL approach. The two approaches are generally recommended for analyses of small studies. We compared the results with an uncorrected logistic model on cases only and a Cox model modified with the Firth correction on cases and non-cases. More details about the performance of the latter model can be found in our earlier work^7^. The aim of our study was to find scenarios of studies on predictive biomarkers that indicate when such studies could be analyzed with a modified case-only model.

## Methods

### Data generation

*N* datasets were generated and all *n* patients within each dataset were assigned to one of four combinations of biological marker *M* (low level: $M=0$; high level: $M=1$) and treatment *T* (standard treatment: $T=0;$ experimental treatment: $T=1$). The probabilities of assignment to each combination depended on the proportion $p_M$ of patients with high marker level, the proportion $p_T$ of patients treated with the experimental treatment, and the odds ratio $\text{ OR}_{MT}$ of the association between marker and treatment^22^.

Event times $t_e$ were generated from a random variable $U_e$ uniformly distributed on the interval [0, 1], *M*, *T*, and the product *MT* of *M* and *T*:

$$\begin{aligned} t_e&=-\frac{\textrm{log} (U_e)}{\lambda _e \textrm{exp} \left( \beta _M M+ \beta _T T+\beta _{I}MT \right) }. \end{aligned}$$

$\text{ exp }\left( \beta _M\right) =\text{ HR}_M$ was the ratio of hazards for high vs. low marker level among patients receiving standard treatment, $\text{ exp }\left( \beta _T\right) =\text{ HR}_T$ was the ratio of hazards for experimental vs. standard treatment among patients with low marker level, $\text{ exp }\left( \beta _{I}\right) =\text{ HR}_{I}$ was the interaction hazard ratio, i.e., the ratio between treatment hazard ratios in high vs. low marker level. An exponential survival distribution with a scale parameter $\lambda _e$ was used to calculate baseline survival with

$$\begin{aligned} \lambda _e=-\frac{1}{t_{end}}\log \left( 1-q_e\right) , \end{aligned}$$

so that before the end of follow-up $t_{end}$ the proportion of patients with low marker level receiving standard treatment who experienced an event was $q_e$, i.e., the exponential survival function $S(t)=\text{ exp }\left( -\lambda _e t\right)$ at $t_{end}$ was $S(t_{end})=1-q_e$. In additional analyses, the baseline survival was calculated with a Weibull survival distribution with increasing or decreasing hazard of event occurrence over time. We do not show these results but refer to them in the discussion. Censoring times $t_c$ were generated similarly from a random uniform variable $U_c,$ scale parameter $\lambda _c$, the proportion $q_c$ of patients with low marker level receiving standard treatment censored before $t_{end}$ (excluding administrative censoring at the end of the study period) and $\beta _M=\beta _T=\beta _{I}=0$ to achieve non-differential censoring by marker and treatment. The patient was specified as experiencing an event at $t_e$ if $t_e< \text{ min }(t_c, t_{end}$) and censored otherwise at $\text{ min }(t_c, t_{end}$).

We generated $N=10000$ datasets with different values for *n* (200, 300, 400, 500, 600, 800, 1000), $p_M$ (0.25, 0.5, 0.75), $\text{ HR}_M$ (0.6, 0.8, 1, 3, 6), $\text{ OR}_{MT}$ (0.5, 1, 2) and $\text{ HR}_{I}$ (0.25, 0.5, 0.75, 1) but only one value of $p_T=0.5$, $q_e=0.2$, $q_c=0.2$, $t_{end}=5$ years and HR$_T=1$. The different specifications were chosen based on real datasets presented and summarized in our earlier work^7^. Briefly, the sample size in these studies varied from 117 to 541. The proportion of patients with high marker levels was 14$\%$, 18$\%$ and about 50$\%$, and the marker effect among patients treated with the standard treatment was either protective ($HR_M$ = 0.67, 0.86) or harmful ($HR_M$ = 3.51, 5.39, 6.60). The ratio between odds of high marker level for patients treated with experimental vs. standard treatment, i.e., $OR_{MT}$, ranged from 0.79 to 2.34, and between 42$\%$ and 58$\%$ of the patients received the experimental treatment. Patients with the low marker level benefitted from the experimental treatment ($HR_T$ between 0.23 and 0.87) and in all studies except one, the benefit of the experimental treatment was greater for patients with high vs. low marker levels ($HR_I$ = 0.08, 0.24, 0.37, 0.63, 1.95). However, since a qualitative interaction between the marker and the treatment is needed to guide treatment choice^23^, we simulated scenarios with equally efficacious treatments among patients with low marker level (HR$_T=1$).

Aggregated data from various clinical studies were used as inputs for the simulation study. All studies were carried out in accordance with relevant guidelines and regulations. All study protocols were approved by responsible institutional committees. The study by de Boo et al.^17^ was approved by the Ethics Committee of the participating medical institutions and the National Agency for Medicines, Finland. The Institutional Review Board at the Helsinki University Hospital, Finland, approved the use of archival tissue for the current translational study. All seven studies in Knauer et al.^18^ had been approved by the respective institutional review boards. The ethical committees of Lund and Linköping universities approved the study by Kok et al.^19^. The study by Schouten et al.^20^ was approved by the Ethical Committee of the University of Heidelberg. The trial described in Vollebergh et al.^21^ was approved by the Institutional Review Board of the Netherlands Cancer Institute. In all those studies, informed consent was obtained from all subjects and/or their legal guardian(s).

### Data analysis

The generated datasets were analyzed using three different models and two parametrizations of each model, and the 95% CIs were calculated according to Wald and PL methods.

A logistic regression of treatment assignment was fitted to case-only data, i.e., *K* patients who experienced an event of interest at times $t_e$ ($e=1,...,K$), using the formula

1 $$\begin{aligned} \text{ log } \frac{\text{ P }(T=1 | t_e, M)}{\text{ P }(T=0 | t_e, M)}=\text{ log } \frac{p_e}{1-p_e} + \gamma _T + \gamma _I M \end{aligned}$$

and

2 $$\begin{aligned} \text{ log } \frac{\text{ P }(T=1 | t_e, M)}{\text{ P }(T=0 | t_e, M)}=\text{ log } \frac{p_e}{1-p_e} + \gamma _{TM_{low}} M_{low} + \gamma _{TM_{high}} M_{high}, \end{aligned}$$

where $\text{ log }(p_e/(1-p_e))$ was a constant (“offset”) term with $p_e$ being the fraction of patients in the full cohort at time $t_e$ assigned to the experimental treatment who were still at risk at time $t_e$. $M_{low}$ and $M_{high}$ were binary variables indicating patients with low and high marker level, respectively. Additionally, models (1) and (2) with modified score functions based on the method developed by Firth^8^ were fitted to case-only data.

A Firth-corrected Cox proportional hazards model was fitted to all generated patients (cases and non-cases) using the hazard function

3 $$\begin{aligned} h(t;T,M)=h_0(t) \text{ exp }\left( \beta _{M} M+ \beta _{T} T+\beta _{I}MT\right) \end{aligned}$$

with baseline hazard function $h_0$ to evaluate the interaction term $\beta _{I}$ and

4 $$\begin{aligned} h(t;T,M)=h_0(t) \text{ exp }\left( {\beta _M M+ \beta _{TM_{low}} TM_{low}+\beta _{TM_{high}} TM_{high}}\right) \end{aligned}$$

to evaluate the treatment effect by marker level, i.e., $\beta _{TM_{low}}$ and $\beta _{TM_{high}}$. $\text{ exp }\left( \beta _{TM_{low}}\right) =\text{ HR}_{TM_{low}}$ and $\text{ exp }\left( \beta _{TM_{high}}\right) =\text{ HR}_{TM_{high}}$ were the hazard ratios for experimental vs. standard treatment in subgroups of low and high marker levels, respectively. $TM_{low}$ and $TM_{high}$ were binary variables defined as $TM_{low}=1$ if $M=0$ and $T=1$, and $TM_{low}=0$ otherwise; $TM_{high}=1$ if $M=1$ and $T=1$, and $TM_{high}=0$ otherwise, to indicate patients receiving experimental treatment in the two marker levels.

As shown by Dai et al.^11^, $\gamma _T \approx \beta _{T}$, $\gamma _I \approx \beta _{I}$, $\gamma _{TM_{low}} \approx \beta _{TM_{low}}$, $\gamma _{TM_{high}} \approx \beta _{TM_{high}}$, when treatment assignment is independent of marker level, censoring is independent of treatment conditionally on marker level and the event is rare for all event times $t_e$. Even though the $\gamma$ parameters are estimated with logistic regressions, they are interpreted as hazard ratios.

As defined by Morris et al.^24^, we calculated several performance measures to summarize estimation of the interaction term across all scenarios, namely (i) bias: $\frac{1}{N_c}\sum _{j=1}^{N_c}\hat{\beta }_{I,j}-\beta _{I}$ or relative bias: $\frac{1}{N_c}\sum _{j=1}^{N_c}\frac{\hat{\beta }_{I,j}-\beta _{I}}{|\beta _{I}|}$, (ii) relative % error in model standard error (ModSE): $100\left( \frac{\widehat{\text{ ModSE }}}{\widehat{\text{ EmpSE }}}-1\right)$ with the model standard error ModSE obtained as $\sqrt{\frac{1}{N_c}\sum _{j=1}^{N_c} \widehat{\text{ Var }}\left( \hat{\beta }_{I,j}\right) }$ and the empirical standard error EmpSE obtained as $\sqrt{\frac{1}{N_c-1}\sum _{j=1}^{N_c}\left( \hat{\beta }_{I,j}-\bar{\beta }_{I}\right) ^2},$ (iii) coverage of the CI: $\frac{1}{N_c}\sum _{j=1}^{N_c} \mathbf{{1}}\left( \hat{\beta }_{l,j}\le \beta _{I}\le \hat{\beta }_{u,j}\right)$ with $\hat{\beta }_{l,j}$ being the lower bound and $\hat{\beta }_{u,j}$ being the upper bound of the 95% CI around $\hat{\beta }_{I,j}$, and (iv) type I error or power: $\frac{1}{N_c}\sum _{j=1}^{N_c} \mathbf{{1}}(p_j\le \alpha )$, where $p_j$ was the p-value obtained with the j-th dataset by testing the null hypothesis $\beta _I=0$ and $\alpha$ was the significance level fixed at 0.05. In all formulas, $N_c$ indicated the number of converged models, $\beta _{I}$ was the true value of the coefficient of the interaction term and $\hat{\beta }_{I,j}$ was the estimate of the interaction coefficient in the j-th dataset. The mean of all $\hat{\beta }_{I,j}$ was indicated as $\bar{\beta }_{I}$ and $\textbf{1}$ was an indicator function. Since the calculations of coverage, type I error and power depended on the CI method, separate calculations were performed for the Wald and PL approach. Additionally, the estimation of treatment effect in the subgroups of low and high marker levels was summarized with the bias and relative percentage error in standard error. For calculations of all performance measures, only datasets with events in at least three combinations of marker and treatment and results from converged models were used. A model was considered converged if the actual number of iterations for a model fit was less than the prespecified maximum number of iterations. However, for summary statistics of the PL-based power and coverage, models with overall convergence and additionally with convergence of the confidence bound were used since the latter is required to determine whether or not the PL confidence interval included zero or the true parameter value.

All simulation scripts were written in R version 4.3.1. The logistf function of the logistf package version 1.26.0^25^ was used to fit a standard logistic and logistic-Firth model. The coxphf function of the coxphf package version 1.13.4^26^ was used with maximally 1000 iterations (maxiter) and a maximum step size (maxstep) of 0.01 to fit a Cox-Firth model. The scripts are available on request from the corresponding author.

**Fig. 1:**
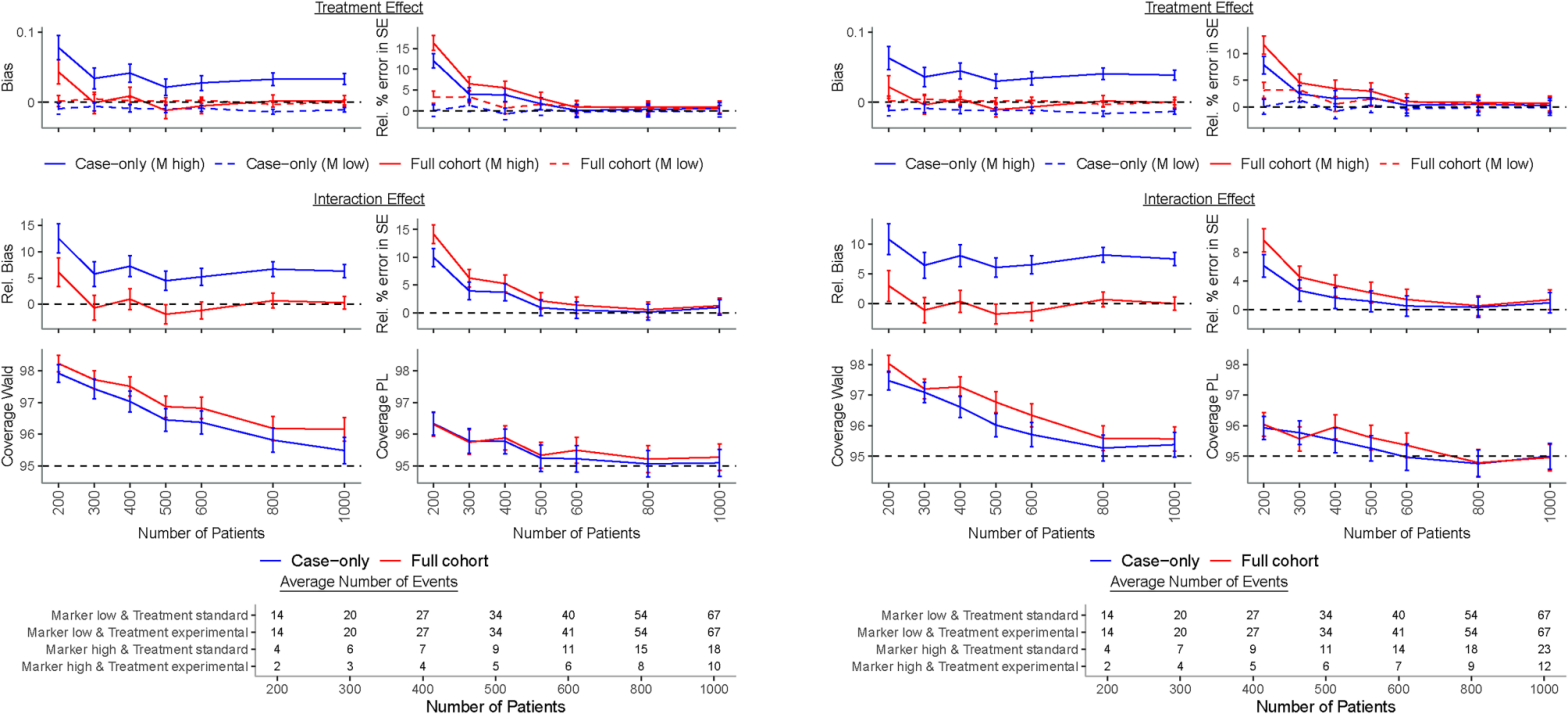
Results of the simulation study for treatment assignment independent of the marker level, i.e., $\text{ OR}_{MT}=1$, and a protective ($\text{ HR}_M=0.8$, left panel) and a null ($\text{ HR}_M=1$, right panel) marker effect among patients treated with the standard treatment. The treatment HRs were $\text{ HR}_{TM_{low}}=1$ and $\text{ HR}_{TM_{high}}=0.5,$ i.e., $\beta _{TM_{low}}=0$ and $\beta _{TM_{high}}=-0.69$, the interaction HR was $\text{ HR}_{I}=0.5$, i.e., $\beta _{I}=-0.69$, and the proportion of patients with high marker level was $p_{M}=0.25$. Case-only results were obtained with a Firth-corrected logistic regression, while full cohort results were obtained with a Firth-corrected Cox proportional hazards model. Number of patients was the number of patients per dataset in full cohort HR, hazard ratio; M, marker; OR, odds ratio; PL, profile likelihood; Rel., relative; SE, standard error.

**Table 1:** Results of the simulation study for treatment assignment independent of the marker level, i.e., $\text{ OR}_{MT}=1$, and a protective ($\text{ HR}_M=0.6$ and $\text{ HR}_M=0.8$) and a null ($\text{ HR}_M=1$) marker effect among patients treated with the standard treatment. The treatment HRs were $\text{ HR}_{TM_{low}}=1$ and $\text{ HR}_{TM_{high}}=0.5,$ i.e., $\beta _{TM_{low}}=0$ and $\beta _{TM_{high}}=-0.69$, the interaction HR was $\text{ HR}_{I}=0.5$, i.e., $\beta _{I}=-0.69$, and the proportion of patients with high marker level was $p_{M}=0.25$. Case-only results were obtained with a Firth-corrected logistic regression, while full cohort results were obtained with a Firth-corrected Cox proportional hazards model.

			Bias						Coverage (%)	Power (%)	
	n	e	$\widehat{\beta }_{TM_{low}}$	SE($\widehat{\beta }_{TM_{low}}$)	$\widehat{\beta }_{TM_{high}}$	SE($\widehat{\beta }_{TM_{high}}$)	$\widehat{\beta }_{I}$	SE($\widehat{\beta }_{I}$)	Wald (PL)	Wald (PL)	N_c_
HR$_M=0.6$											
Case-only	200	31	0	−0.1	0.1	17.3	15.1	15.0	98.2 ( 96.7 )	1.5 ( 5.9 )	9849
	400	62	0	$-$0.8	0	6.3	6.9	6.1	97.4 ( 96.1 )	5.5 ( 11.2 )	10000
	600	94	0	$-$0.3	0	1.4	4.1	1.3	96.6 ( 95.2 )	12.2 ( 16.9 )	10000
Full cohort	200	31	0	3.5	0.1	22	9.9	19.4	98.6 ( 96.7 )	1.7 ( 6.3 )	9830
	400	62	0	0.8	0	8.4	1.7	8.0	97.9 ( 96.2 )	6.2 ( 12.4 )	9986
	600	94	0	1.1	0	2.2	$-$0.8	2.2	97.2 ( 95.6 )	13.7 ( 18.7 )	10000
HR$_M=0.8$											
Case-only	200	34	0	0	0.1	12.0	12.6	10.0	97.9 ( 96.3 )	2.7 ( 7.7 )	9966
	400	65	0	$-$0.8	0	3.8	7.2	3.7	97.0 ( 95.8 )	9.3 ( 13.8 )	10000
	600	98	0	$-$0.3	0	0.1	5.2	0.5	96.4 ( 95.2 )	17.0 ( 20.6 )	10000
Full cohort	200	34	0	3.3	0	16.3	6.1	14.2	98.2 ( 96.3 )	2.9 ( 8.6 )	9955
	400	65	0	0.6	0	5.5	1.0	5.2	97.5 ( 95.9 )	10.1 ( 15.6 )	9997
	600	98	0	1.1	0	0.8	$-$1.2	1.4	96.8 ( 95.5 )	19.4 ( 23.5 )	10000
HR$_M=1$											
Case-only	200	34	0	0.1	0.1	7.8	10.8	6.1	97.5 ( 95.9 )	4.2 ( 9.3 )	9990
	400	68	0	$-$0.8	0	1.6	8.0	1.6	96.6 ( 95.5 )	12.8 ( 16.1 )	10000
	600	102	0	$-$0.3	0	0.3	6.5	0.5	95.7 ( 95.0 )	20.9 ( 23.8 )	10000
Full cohort	200	34	0	3.1	0	11.5	3.0	9.7	98.0 ( 96.0 )	4.3 ( 10.3 )	9983
	400	68	0	0.6	0	3.4	0.4	3.4	97.3 ( 96.0 )	14.0 ( 18.4 )	9998
	600	102	0	1.0	0	1.0	$-$1.4	1.4	96.3 ( 95.4 )	23.8 ( 27.4 )	10000

Bias for $\widehat{\beta }_{TM_{low}}$, $\widehat{\beta }_{TM_{high}}$ and relative bias (%) for SE($\widehat{\beta }_{TM_{low}}$), SE($\widehat{\beta }_{TM_{high}}$), $\widehat{\beta }_{I}$, SE($\widehat{\beta }_{I})$

Other parameters: $$\text{ OR}_{MT}=1$$, $$\text{ HR}_{TM_{low}}=1$$, $$\text{ HR}_{TM_{high}}=0.5$$, $$\text{ HR}_{I}=0.5$$, $$p_{M}=0.25$$

e, average number of events per dataset; HR, hazard ratio; n, number of patients per dataset in full cohort; N_c_, number of converged models; OR, odds ratio; PL, profile likelihood; SE, standard error

## Simulation results

**Fig. 2:**
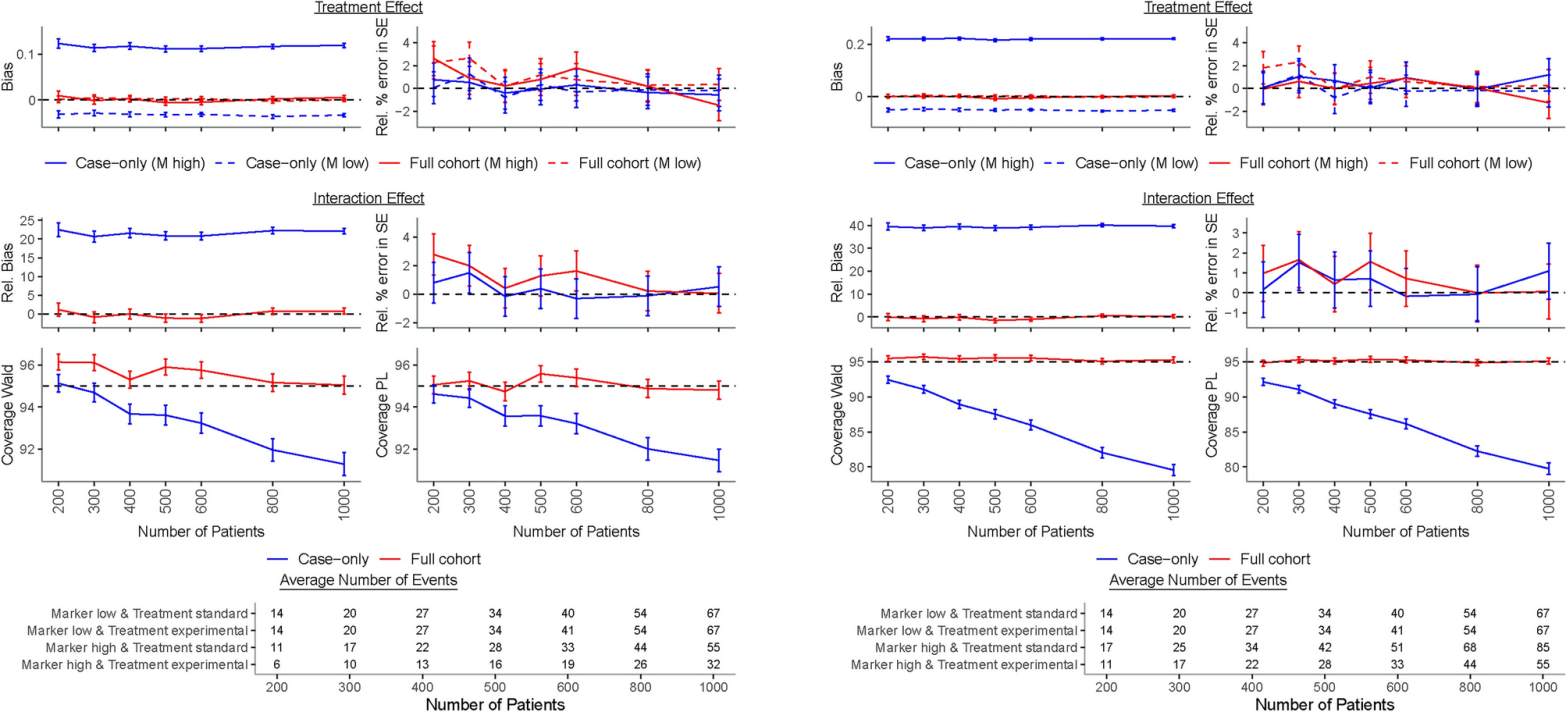
Results of the simulation study for treatment assignment independent of the marker level, i.e., $\text{ OR}_{MT}=1$, and a harmful ($\text{ HR}_M=3$, left panel; $\text{ HR}_M=6$, right panel) marker effect among patients treated with the standard treatment. The treatment HRs were $\text{ HR}_{TM_{low}}=1$ and $\text{ HR}_{TM_{high}}=0.5,$ i.e., $\beta _{TM_{low}}=0$ and $\beta _{TM_{high}}=-0.69$, the interaction HR was $\text{ HR}_{I}=0.5$, i.e., $\beta _{I}=-0.69$, and the proportion of patients with high marker level was $p_{M}=0.25$. Case-only results were obtained with a Firth-corrected logistic regression, while full cohort results were obtained with a Firth-corrected Cox proportional hazards model. Number of patients was the number of patients per dataset in full cohort HR, hazard ratio; M, marker; OR, odds ratio; PL, profile likelihood; Rel., relative; SE, standard error.

Under marker-treatment independence, i.e., $\text{ OR}_{MT}=1$, the interaction and the treatment effect coefficients and their standard errors estimated with the Firth-corrected case-only method showed usually an acceptable bias when the marker was protective or null among patients treated with the standard treatment, i.e., $\text{ HR}_M \le 1$ (Fig. 1, Table 1). The event rate in these scenarios was such that 10–20$\%$ of patients experienced an event over the 5-year follow-up period and the type I error for the interaction coefficient was around or slightly below 5$\%$ for both the Wald and PL method (see Supplementary Table 3 for an example scenario). For harmful markers, however, the interaction coefficient was heavily positively biased with bias up to 50% (data not shown), irrespective of sample size. This bias came from a negative bias of the treatment effect among low marker level patients and a positive bias among high marker level patients (Fig. 2, Table 2). Even a small bias in the treatment coefficient for the two marker levels led to a large relative bias for the interaction coefficient because the estimated treatment coefficient in the low marker level was negative instead of 0 and in the high marker level was away from the true value and towards 0. That caused that the interaction effect was away from the truth and also towards 0. The event rate for harmful markers was always larger so that 20-55$\%$ of patients experienced an event over the 5-year follow-up period, and the stronger the marker effect the larger the event rate and the larger the bias. If treatment assignment depended on marker level, i.e., $\text{ OR}_{MT}\ne 1$, the interaction coefficient and the treatment effect coefficient in the high marker level were severely biased with the direction of bias related to the direction of dependence (Supplementary Fig. 1–2, Supplementary Table 1–2) and the type I error was substantially above the nominal level of 5$\%$.

Convergence of the Firth-corrected case-only model was very high for all scenarios. Coverage was often above the nominal level of 95% and approached 95% with increasing sample size when $\text{ HR}_M\le 1$ and $\text{ OR}_{MT}=1$. It was usually closer to the nominal level when it was calculated with the PL in comparison to the Wald approach. However, coverage for both methods was below the nominal level and moved away from nominal level with larger sample size when $\text{ HR}_M>1$ or $\text{ OR}_{MT} \ne 1$, i.e., when the interaction coefficient but not its standard error was biased. This often led to 95% CIs for the interaction coefficient which did not include its true value (Fig. 1–2, Supplementary Fig. 1–2, Tables 1–2, Supplementary Table 1–2). Moreover, statistical power also depended strongly on the marker-treatment association and it decreased with larger values of the $\text{ OR}_{MT}$. Under marker-treatment independence, power was lower than 80% for sample sizes smaller than 600 with event rates over 5 years below 20% when the marker was protective or null and was consistently slightly higher for PL-based vs. Wald-based CI (Tables 1–2, Supplementary Table 1–2).

As previously shown^7^, the full cohort analysis with the Firth-corrected Cox model was virtually unbiased for sample sizes down to 200 and overall event rates over 5 years above 20$\%$ when the marker was harmful among patients treated with the standard treatment, i.e., $\text{ HR}_M>1$. Otherwise, the interaction coefficient and its standard error were substantially biased. The interaction coefficient was biased towards and away from the null and standard error was overestimated for small sample sizes, but bias decreased and monotonically approached zero as the sample size increased. These results did not depend on the marker-treatment association nor the censoring rate.

Under marker-treatment independence, coverage, power and estimation of the standard error of the interaction coefficient were similarly good for the case-only analysis with the logistic-Firth model and the full cohort Firth-corrected Cox model for protective or null markers, i.e., $\text{ HR}_M \le 1$. However, relative bias of the interaction coefficient persists at 5-10$\%$ with the case-only model regardless of sample size and is lower, often around or below 5$\%$ for the full cohort Firth-corrected Cox model. Power is generally low at 600 patients or less for either method (Fig. 1, Table 1).

It is noteworthy that the Firth-correction improved the performance of the case-only analysis in general. When $\text{ HR}_M\le 1$ and sample size was small, relative bias of all evaluated coefficients decreased when the correction was used in comparison to a standard case-only model without the correction (Supplementary Fig. 3). However, the standard Firth correction shrinks all parameters, including the intercept, and therefore produces estimates which are slightly biased at 5$\%$ or less even for large sample sizes.

**Table 2:** Results of the simulation study for treatment assignment independent of the marker level, i.e., $\text{ OR}_{MT}=1$, and a harmful ($\text{ HR}_M=3$ and $\text{ HR}_M=6$) marker effect among patients treated with the standard treatment. The treatment HRs were $\text{ HR}_{TM_{low}}=1$ and $\text{ HR}_{TM_{high}}=0.5$, i.e., $\beta _{TM_{low}}=0$ and $\beta _{TM_{high}}=-0.69$, the interaction HR was $\text{ HR}_{I}=0.5$, i.e., $\beta _{I}=-0.69$, and the proportion of patients with high marker level was $p_{M}=0.25$. Case-only results were obtained with a Firth-corrected logistic regression, while full cohort results were obtained with a Firth-corrected Cox proportional hazards model.

			Bias						Coverage (%)	Power (%)	
	n	e	$\widehat{\beta }_{TM_{low}}$	SE($\widehat{\beta }_{TM_{low}}$)	$\widehat{\beta }_{TM_{high}}$	SE($\widehat{\beta }_{TM_{high}}$)	$\widehat{\beta }_{I}$	SE($\widehat{\beta }_{I}$)	Wald (PL)	Wald (PL)	N_c_
HR$_M=3$											
Case-only	200	45	0	0.1	0.1	0.8	22.5	0.8	95.1 ( 94.6 )	11.0 ( 12.8 )	10000
	400	89	0	$-$0.8	0.1	$-$0.4	21.6	$-$0.2	93.7 ( 93.6 )	21.4 ( 22.6 )	10000
	600	133	0	$-$0.3	0.1	0.3	20.8	$-$0.3	93.2 ( 93.2 )	31.8 ( 32.7 )	10000
Full cohort	200	45	0	2.3	0	2.6	1.1	2.8	96.2 ( 95.1 )	15.6 ( 18.6 )	10000
	400	89	0	0.2	0	0.2	0	0.4	95.3 ( 94.7 )	32.4 ( 34.2 )	10000
	600	133	0	0.8	0	1.8	$-$1.2	1.6	95.8 ( 95.4 )	47.3 ( 48.8 )	10000
HR$_M=6$											
Case-only	200	56	$-$0.1	0	0.2	0.1	39.5	0.2	92.4 ( 92.1 )	10.8 ( 11.6 )	10000
	400	110	$-$0.1	$-$0.8	0.2	0.7	39.6	0.6	88.9 ( 89.0 )	18.2 ( 18.7 )	10000
	600	165	$-$0.1	$-$0.2	0.2	0.9	39.2	$-$0.2	86.0 ( 86.2 )	26.1 ( 26.5 )	10000
Full cohort	200	56	0	1.8	0	$-$0.1	$-$0.1	1.0	95.5 ( 94.8 )	22.2 ( 24.0 )	10000
	400	110	0	$-$0.1	0	0	$-$0.2	0.4	95.4 ( 95.1 )	42.1 ( 43.2 )	10000
	600	165	0	0.6	0	0.9	$-$1.1	0.7	95.5 ( 95.2 )	59.6 ( 60.2 )	10000

Bias for $\widehat{\beta }_{TM_{low}}$, $\widehat{\beta }_{TM_{high}}$ and relative bias (%) for SE($\widehat{\beta }_{TM_{low}}$), SE($\widehat{\beta }_{TM_{high}}$), $\widehat{\beta }_{I}$, SE($\widehat{\beta }_{I}$)

Other parameters: $$\text{ OR}_{MT}=1$$, $$\text{ HR}_{TM_{low}}=1$$, $$\text{ HR}_{TM_{high}}=0.5$$, $$\text{ HR}_{I}=0.5$$, $$p_{M}=0.25$$

e, average number of events per dataset; HR, hazard ratio; n, number of patients per dataset in full cohort; N_c_, number of converged models; OR, odds ratio; PL, profile likelihood; SE, standard error

## Discussion

A modified case-only model can be used to analyze relatively small studies of predictive markers when the overall event rate is low, i.e., when the event is rare at baseline and the marker is protective, and when the treatment assignment is independent from the marker level, e.g., patients are randomized to treatment. In such studies, the model estimates the interaction and treatment coefficient and their standard errors with acceptable bias. Moreover, the coverage of the CI for the interaction coefficient is at or just slightly above the nominal level while type I error is at or slightly below the nominal level. The number of biomarker measurements and corresponding costs are reduced by 80$\%$ or more compared with a full cohort analysis.

However, the modified case-only model has to be used cautiously since our simulation results are based on a finite series of scenarios derived from previous clinical studies of breast cancer. Moreover, model performance appears to be sensitive to the assumptions. The model should not be used when the event rate is not low, e.g., due to a harmful marker, or when treatment assignment depends on the marker level.

Most clinical studies on treatment heterogeneity with failure time endpoints are analyzed using data from a cohort and applying a Cox regression with a multiplicative interaction term between marker and treatment^6^. In studies with a small number of patients, unbiased results of a Cox regression are guaranteed when the marker is harmful among patients treated with the standard treatment. The model can yield biased results when the marker is protective or null in this subgroup of patients. In our earlier work^7^, we showed that the bias is reduced when the score function of a Cox model is modified using a Firth correction. In the current study, we show that bias reduction can also be obtained by analyzing only patients who experience the survival event of interest using a Firth corrected case-only model. The Firth-corrected model with the full cohort and with cases only, show acceptable performance only when there is no association between the marker level and the treatment assignment. When there is a dependence between the marker and the treatment or the marker is harmful among patients treated with the standard treatment (leading to a high event rate), a Firth-corrected case-only model is severely biased. Thus, our study confirms the importance of assumptions for valid results of the case-only approach discussed in the literature, namely, a low event rate and marker-treatment independence^10,11,16^.

The comparison between results obtained with a standard Cox model and a standard case-only model for survival outcomes has been previously conducted using randomized studies, where the independence between the two factors that interact with each other is established by design^10,11^. However, in epidemiological studies, it has been shown that a dependence causes bias^16^. Even in retrospective data from a randomized clinical trial, independence between marker and treatment is not guaranteed. For example, the availability of tissue for biomarker measurements may depend on marker or treatment. If the dependence, on the other hand, can be explained by a third factor, the bias can be reduced or eliminated by adjusting the case-only model for this third factor^27^. We did not evaluate this in the simulation study, since none of the patient and tumor characteristics in the BC example studies explained the dependence between the marker and the treatment.

Our simulation study does not address complex situations that may occur in some cancer studies. For example, risk of relapse or death can increase over time or the baseline hazard changes in other ways, i.e., hazards are not constant. Although some limited sensitivity analyses indicated that our results do hold in more complex situations, e.g., when a non-constant hazard at baseline was used instead of the exponential hazard, caution needs to be used when applying the case-only design to situations not evaluated here.

An important advantage of using a case-only approach in a retrospective study is the cost reduction since marker measurements are only performed for a subset of trial participants. With the resources for a full cohort study, one could pool patients with events from multiple trials, which would lead to increased power. However, the case-only approach estimates the marker-treatment interaction and the treatment effects by marker level but not the marker effect. If the latter is an objective, a full cohort or an augmented case-only design is needed. The augmented case-only design is a hybrid method which combines case-only and case-control designs by randomly sampling controls from both treatment arms or from the experimental treatment only^11^.

The assumptions under which the case-only design can be useful are not easy to verify prior to study onset. However, the expected event rate is generally known during the design phase of a study and the independence assumption is per definition fulfilled in randomized designs, making a large number of studies suitable for retrospective case-only analyses. The direction of the marker effect has to be known from previous studies. Since it cannot be estimated with a case-only model, it is not even known after the study. Many predictive marker candidates were, however, previously used as prognostic markers. Noteworthy, results with acceptable bias for a case-only model with a harmful marker cannot be obtained by simply recoding and estimating $1/\text{HR}_M$ for the standard treatment. Changing the reference category for the marker automatically recodes the interaction effect to $1/\text{HR}_I$. Although, the different combinations of marker and treatment are shuffled and the comparison groups are different, the event rate in the different subgroups which eventually influences the bias is not changed.

In conclusion, we show that small studies on predictive markers can be analyzed with a case-only model when the event rate is low, treatment assignment is independent from marker level and the marker is protective or null among patients who received the standard treatment. The design offers substantial cost savings.

## Supplementary Information

Supplementary Information.

## Data Availability

Computer scripts in the programming language R are available on request from the corresponding author.
