# Supplementary material for: Case-only analysis in small studies of predictive biomarkers
Source: Sci Rep. 2025 Apr 16;15:13068. doi: 10.1038/s41598-025-96904-9 (PMC12003872; doi:10.1038/s41598-025-96904-9)
Supplement: Supplementary file 1 — Supplementary Information. [file 41598_2025_96904_MOESM1_ESM.pdf]

Supplementary Material

Case-only analysis in small studies of predictive biomarkers

Hauptmann M[1,*], Nguyen VH[1,**], Sollfrank L[1], Linn SC[2,3,4], Jóźwiak K[1]

[1]Institute of Biostatistics and Registry Research, Brandenburg Medical School Theodor Fontane, Neuruppin, Germany

[2]Division of Molecular Pathology, The Netherlands Cancer Institute, Amsterdam, The Netherlands

[3]Department of Medical Oncology, The Netherlands Cancer Institute, Amsterdam, The Netherlands

[4]Department of Pathology, University Medical Center, Utrecht, The Netherlands

[*]Corresponding author; Corresponding address: Fehrbelliner Straße 39, 16816 Neuruppin, Germany, michael.hauptmann@mhb-fontane.de

[**]Now at Leibniz Centre for Agricultural Landscape Research (ZALF), Müncheberg, Germany

Figure 1: Results of the simulation study for treatment assignment dependent of the marker level, i.e., $\mathrm{OR}_{MT} = 2$ and a protective ($\mathrm{HR}_M = 0.8$, left panel) and a harmful ($\mathrm{HR}_M = 3$, right panel) marker effect among patients treated with the standard treatment. The treatment HRs were $\mathrm{HR}_{TM_{low}} = 1$ and $\mathrm{HR}_{TM_{high}} = 0.5$, i.e., $\beta_{TM_{low}} = 0$ and $\beta_{TM_{high}} = -0.69$, the interaction HR was $\mathrm{HR}_I = 0.5$, i.e., $\beta_I = -0.69$, and the proportion of patients with high marker level was $p_M = 0.25$. Case-only results were obtained with a Firth-corrected logistic regression, while full cohort results were obtained with a Firth-corrected Cox proportional hazards model.

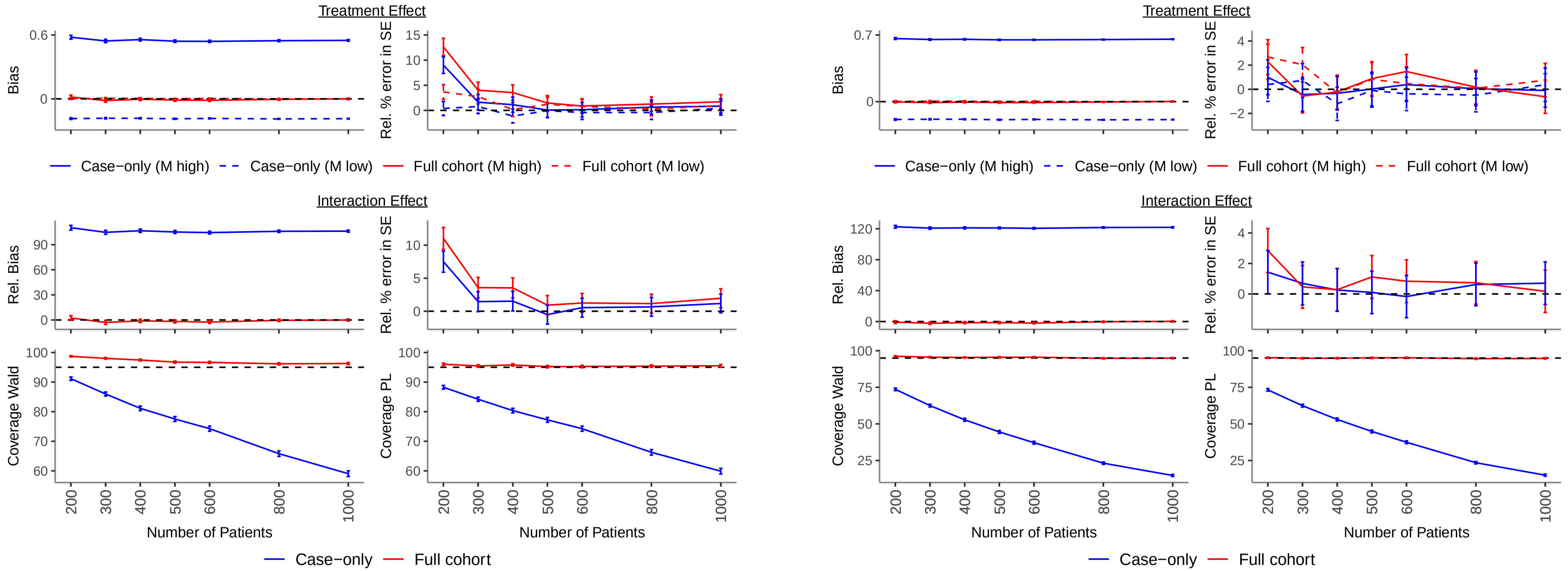

Average Number of Events (left panel)

	200	300	400	500	600	800	1000
Marker low & Treatment standard	15	22	29	37	44	59	73
Marker low & Treatment experimental	12	19	25	31	37	49	62
Marker high & Treatment standard	3	4	5	7	8	11	14
Marker high & Treatment experimental	2	4	5	6	7	10	12

Number of Patients

Average Number of Events (right panel)

	200	300	400	500	600	800	1000
Marker low & Treatment standard	15	22	29	37	44	59	73
Marker low & Treatment experimental	12	19	25	31	37	49	62
Marker high & Treatment standard	8	12	16	21	25	33	41
Marker high & Treatment experimental	8	12	16	20	24	32	40

Number of Patients

Number of patients was the number of patients per dataset in full cohort
HR, hazard ratio; M, marker; OR, odds ratio; PL, profile likelihood; Rel., relative; SE, standard error

Figure 2: Results of the simulation study for treatment assignment dependent of the marker level, i.e., $\mathrm{OR}_{MT} = 0.5$, and a protective ($\mathrm{HR}_M = 0.8$, left panel) and a harmful ($\mathrm{HR}_M = 3$, right panel) marker effect among patients treated with the standard treatment. The treatment HRs were $\mathrm{HR}_{TM_{low}} = 1$ and $\mathrm{HR}_{TM_{high}} = 0.5$, i.e., $\beta_{TM_{low}} = 0$ and $\beta_{TM_{high}} = -0.69$, the interaction HR was $\mathrm{HR}_I = 0.5$, i.e., $\beta_I = -0.69$, and the proportion of patients with high marker level was $p_M = 0.25$. Case-only results were obtained with a Firth-corrected logistic regression, while full cohort results were obtained with a Firth-corrected Cox proportional hazards model.

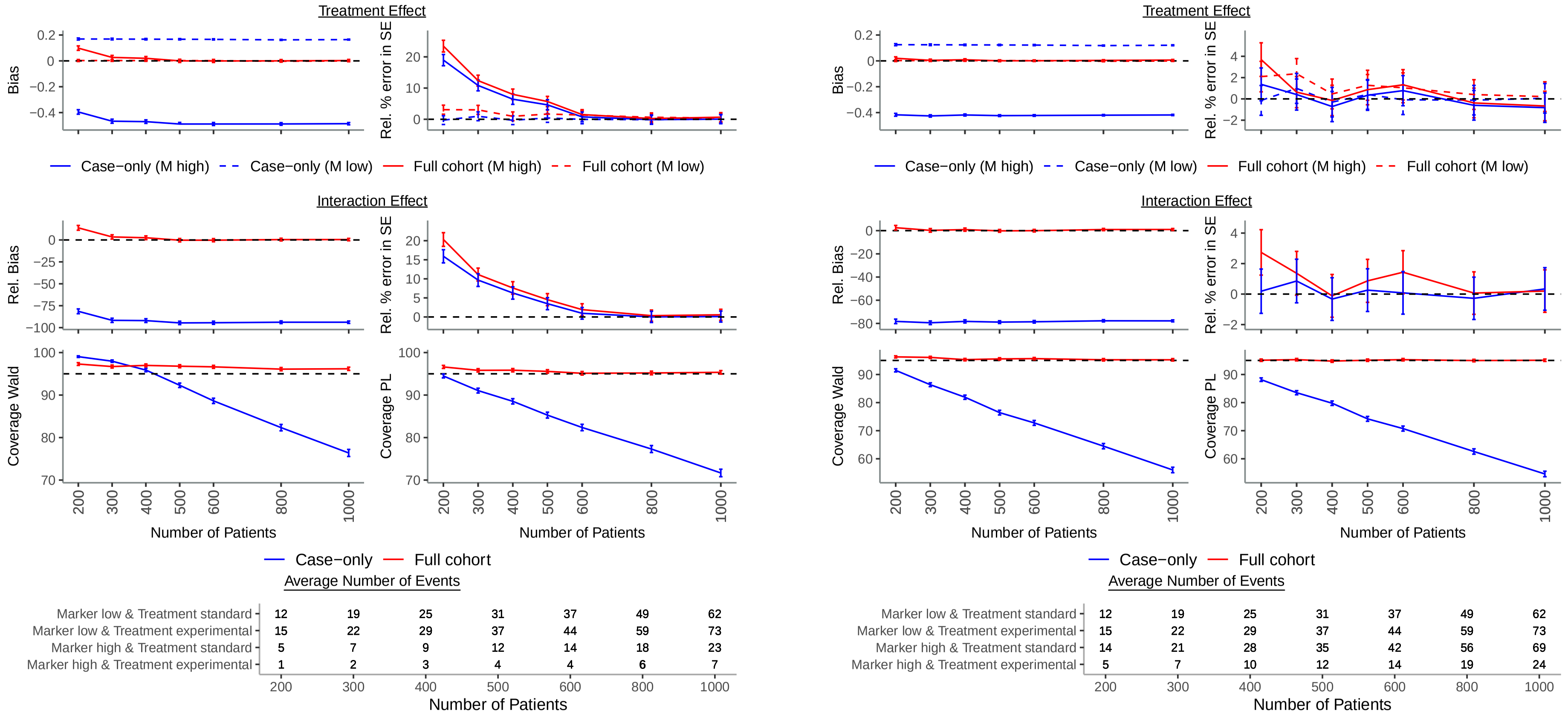

Number of patients was the number of patients per dataset in full cohort

HR, hazard ratio; M, marker; OR, odds ratio; PL, profile likelihood; Rel., relative; SE, standard error

Figure 3: Results of the simulation study for treatment assignment independent of the marker level, i.e., $\text{OR}_{MT} = 1$, and a protective ($\text{HR}_M = 0.6$, left panel; $\text{HR}_M = 0.8$, right panel) marker effect among patients treated with the standard treatment. The treatment HRs were $\text{HR}_{TM_{low}} = 1$ and $\text{HR}_{TM_{high}} = 0.5$, i.e., $\beta_{TM_{low}} = 0$ and $\beta_{TM_{high}} = -0.69$, the interaction HR was $\text{HR}_I = 0.5$, i.e., $\beta_I = -0.69$, and the proportion of patients with high marker level was $p_M = 0.25$. Case-only results were obtained with a Firth-corrected or an uncorrected logistic regression.

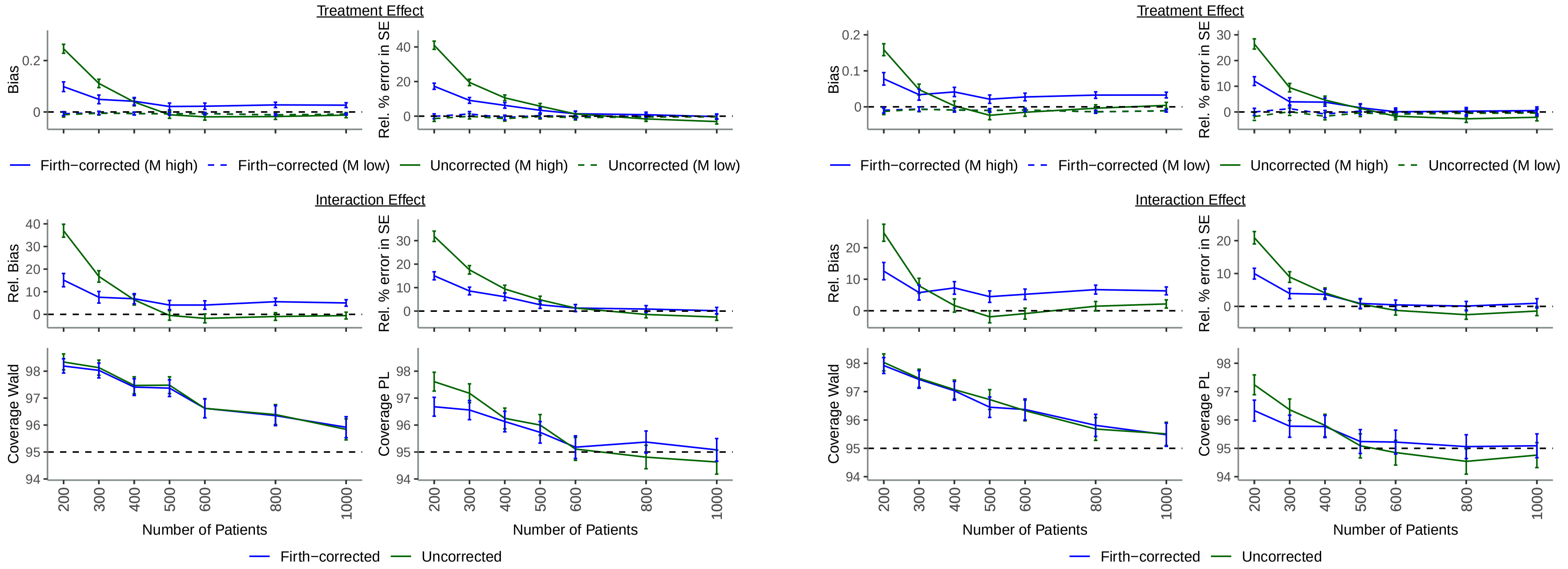

Average Number of Events

	200	300	400	500	600	800	1000
Marker low & Treatment standard	14	20	27	34	40	54	67
Marker low & Treatment experimental	14	20	27	34	41	54	67
Marker high & Treatment standard	3	4	6	7	8	11	14
Marker high & Treatment experimental	1	2	3	4	4	6	7

Number of Patients

Average Number of Events

	200	300	400	500	600	800	1000
Marker low & Treatment standard	14	20	27	34	40	54	67
Marker low & Treatment experimental	14	20	27	34	41	54	67
Marker high & Treatment standard	4	6	7	9	11	15	18
Marker high & Treatment experimental	2	3	4	5	6	8	10

Number of Patients

Number of patients was the number of patients per dataset in full cohort
HR, hazard ratio; M, marker; OR, odds ratio; PL, profile likelihood; Rel., relative; SE, standard error

Table 1: Results of the simulation study for treatment assignment dependent of the marker level, i.e., $\mathrm{OR}_{MT} = 2$, and a protective ($\mathrm{HR}_M = 0.8$) and a harmful ($\mathrm{HR}_M = 3$) marker effect among patients treated with the standard treatment. The treatment HRs were $\mathrm{HR}_{TM_{low}} = 1$ and $\mathrm{HR}_{TM_{high}} = 0.5$, i.e., $\beta_{TM_{low}} = 0$ and $\beta_{TM_{high}} = -0.69$, the interaction HR was $\mathrm{HR}_I = 0.5$, i.e., $\beta_I = -0.69$, and the proportion of patients with high marker level was $p_M = 0.25$. Case-only results were obtained with a Firth-corrected logistic regression, while full cohort results were obtained with a Firth-corrected Cox proportional hazards model.

			Bias*						Coverage (%)	Power (%)	
	n	e	$\widehat{\beta}_{TM_{low}}$	$\mathrm{SE}(\widehat{\beta}_{TM_{low}})$	$\widehat{\beta}_{TM_{high}}$	$\mathrm{SE}(\widehat{\beta}_{TM_{high}})$	$\widehat{\beta}_I$	$\mathrm{SE}(\widehat{\beta}_I)$	Wald (PL)	Wald (PL)	N_c
$\mathrm{HR}_M = 0.8$											
Case-only	200	32	-0.2	0.4	0.6	9.0	110.3	7.5	91.2 (88.2)	1.0 (3.7)	9944
	400	64	-0.2	-1.1	0.6	1.1	106.7	1.5	81.1 (80.4)	2.5 (4.2)	10000
	600	96	-0.2	-0.4	0.5	0.1	104.5	0.5	74.3 (74.2)	3.5 (4.8)	10000
Full cohort	200	32	0	3.7	0	12.6	2.4	11.0	98.7 (96.0)	5.3 (9.8)	9942
	400	64	0	0.2	0	3.5	-1.1	3.5	97.4 (95.8)	14.5 (17.3)	9982
	600	96	0	0.8	0	0.9	-2.5	1.2	96.6 (95.3)	22.9 (24.6)	9999
$\mathrm{HR}_M = 3$											
Case-only	200	43	-0.2	0.4	0.7	1.0	122.6	1.4	73.6 (73.2)	4.1 (5.0)	10000
	400	86	-0.2	-1.2	0.7	-0.3	121.1	0.3	52.8 (53.0)	5.8 (6.1)	10000
	600	130	-0.2	-0.4	0.6	0.4	120.5	-0.2	37.1 (37.4)	6.6 (6.9)	10000
Full cohort	200	43	0	2.7	0	2.3	-0.7	2.8	96.2 (95.3)	17.9 (19.3)	10000
	400	86	0	-0.3	0	-0.2	-1.4	0.3	95.4 (94.9)	34.0 (34.7)	10000
	600	130	0	0.5	0	1.5	-2.0	0.8	95.6 (95.2)	48.8 (49.1)	10000

*Bias for $\widehat{\beta}_{TM_{low}}$, $\widehat{\beta}_{TM_{high}}$ and relative bias (%) for $\mathrm{SE}(\widehat{\beta}_{TM_{low}})$, $\mathrm{SE}(\widehat{\beta}_{TM_{high}})$, $\widehat{\beta}_I$, $\mathrm{SE}(\widehat{\beta}_I)$

Other parameters: $\mathrm{OR}_{MT} = 2$, $\mathrm{HR}_{TM_{low}} = 1$, $\mathrm{HR}_{TM_{high}} = 0.5$, $\mathrm{HR}_I = 0.5$, $p_M = 0.25$

e, average number of events per dataset; HR, hazard ratio; n, number of patients per dataset in full cohort; N_c, number of converged models; OR, odds ratio; PL, profile likelihood; SE, standard error

Table 2: Results of the simulation study for treatment assignment dependent of the marker level, i.e., $OR_{MT} = 0.5$, and a protective ($HR_M = 0.8$) and a harmful ($HR_M = 3$) marker effect among patients treated with the standard treatment. The treatment HRs were $HR_{TM_{low}} = 1$ and $HR_{TM_{high}} = 0.5$, i.e., $\beta_{TM_{low}} = 0$ and $\beta_{TM_{high}} = -0.69$, the interaction HR was $HR_I = 0.5$, i.e., $\beta_I = -0.69$, and the proportion of patients with high marker level was $p_M = 0.25$. Case-only results were obtained with a Firth-corrected logistic regression, while full cohort results were obtained with a Firth-corrected Cox proportional hazards model.

			Bias*						Coverage (%)	Power (%)	
	n	e	$\widehat{\beta}_{TM_{low}}$	SE($\widehat{\beta}_{TM_{low}}$)	$\widehat{\beta}_{TM_{high}}$	SE($\widehat{\beta}_{TM_{high}}$)	$\widehat{\beta}_I$	SE($\widehat{\beta}_I$)	Wald (PL)	Wald (PL)	N_c
$HR_M = 0.8$											
Case-only	200	33	0.2	-0.3	-0.4	19.0	-81.4	15.9	99.0 (94.5)	10.9 (23.5)	9975
	400	66	0.2	-0.3	-0.5	6.4	-92.0	6.3	95.9 (88.5)	39.2 (47.7)	10000
	600	99	0.2	0	-0.5	0.8	-94.4	1.0	88.6 (82.4)	62.2 (66.3)	10000
Full cohort	200	33	0	3.1	0.1	23.4	13.8	20.3	97.3 (96.6)	1.1 (5.8)	9954
	400	66	0	0.9	0	8.0	2.5	7.6	97.0 (95.8)	5.4 (12.8)	9999
	600	99	0	1.4	0	1.5	-0.3	1.9	96.6 (95.1)	13.0 (20.0)	10000
$HR_M = 3$											
Case-only	200	46	0.1	-0.1	-0.4	1.4	-78.2	0.2	91.5 (88.2)	43.9 (47.5)	10000
	400	92	0.1	-0.3	-0.4	-0.7	-78.2	-0.3	81.9 (79.8)	77.1 (78.3)	10000
	600	137	0.1	-0.1	-0.4	0.8	-78.6	0.1	72.8 (70.8)	92.0 (92.3)	10000
Full cohort	200	46	0	2.1	0	3.7	2.5	2.7	96.3 (95.1)	12.2 (16.6)	10000
	400	92	0	0.5	0	-0.1	0.9	-0.1	95.3 (94.8)	28.5 (31.9)	10000
	600	137	0	1.0	0	1.3	0	1.4	95.7 (95.3)	42.5 (45.1)	10000

*Bias for $\widehat{\beta}_{TM_{low}}$, $\widehat{\beta}_{TM_{high}}$ and relative bias (%) for SE($\widehat{\beta}_{TM_{low}}$), SE($\widehat{\beta}_{TM_{high}}$), $\widehat{\beta}_I$, SE($\widehat{\beta}_I$)
Other parameters: $OR_{MT} = 0.5$, $HR_{TM_{low}} = 1$, $HR_{TM_{high}} = 0.5$, $HR_I = 0.5$, $p_M = 0.25$
e, average number of events per dataset; HR, hazard ratio; n, number of patients per dataset in full cohort; N_c, number of converged models; OR, odds ratio; PL, profile likelihood; SE, standard error

Table 3: Results of the simulation study for treatment assignment independent of the marker level, i.e., $\mathrm{OR}_{MT} = 1$, and a protective ($\mathrm{HR}_M = 0.8$) marker effect among patients treated with the standard treatment. The treatment HRs were $\mathrm{HR}_{TM_{low}} = 1$ and $\mathrm{HR}_{TM_{high}} = 1$, i.e., $\beta_{TM_{low}} = 0$ and $\beta_{TM_{high}} = 0$, the interaction HR was $\mathrm{HR}_I = 1$, i.e., $\beta_I = 0$, and the proportion of patients with high marker level was $p_M = 0.25$. Case-only results were obtained with a Firth-corrected logistic regression, while full cohort results were obtained with a Firth-corrected Cox proportional hazards model.

			Bias*						Coverage (%)	Type I error (%)	
	n	e	$\widehat{\beta}_{TM_{low}}$	SE($\widehat{\beta}_{TM_{low}}$)	$\widehat{\beta}_{TM_{high}}$	SE($\widehat{\beta}_{TM_{high}}$)	$\widehat{\beta}_I$	SE($\widehat{\beta}_I$)	Wald (PL)	Wald (PL)	N_c
Case-only	200	34	0	0	0	3.6	0	2.8	980. (95.6)	2.0 (4.4)	9994
	400	69	0	-0.8	0	0.3	0	0.3	96.4 (95.2)	3.6 (4.8)	10000
	600	103	0	-0.3	0	0.6	0	0.6	96.3 (95.4)	3.7 (4.6)	10000
Full cohort	200	34	0	3.1	0	7.5	0	6.4	97.9 (95.4)	2.1 (4.6)	9968
	400	69	0	0.5	0	2.3	0	2.1	96.7 (95.5)	3.3 (4.5)	9996
	600	103	0	1.0	0	1.4	0	1.8	96.3 (95.5)	3.7 (4.5)	10000

*Bias for $\widehat{\beta}_{TM_{low}}$, $\widehat{\beta}_{TM_{high}}$ and relative bias (%) for SE($\widehat{\beta}_{TM_{low}}$), SE($\widehat{\beta}_{TM_{high}}$), $\widehat{\beta}_I$, SE($\widehat{\beta}_I$)

Other parameters: $\mathrm{OR}_{MT} = 0.5$, $\mathrm{HR}_{TM_{low}} = 1$, $\mathrm{HR}_{TM_{high}} = 0.5$, $\mathrm{HR}_I = 0.5$, $p_M = 0.25$

e, average number of events per dataset; HR, hazard ratio; n, number of patients per dataset in full cohort; N_c, number of converged models; OR, odds ratio; PL, profile likelihood; SE, standard error
